# Supplementary material for: Quantifying the impact of COVID-19 on e-bike safety in China via multi-output and clustering-based regression models
Source: PLoS One. 2021 Aug 20;16(8):e0256610. doi: 10.1371/journal.pone.0256610 (PMC8378728; doi:10.1371/journal.pone.0256610)
Supplement: S1 File — (ZIP) [file pone.0256610.s001.zip › í╛Supporting Informationí┐Impact of COVID-19 on E-Bike Safety/Data_Brief.pdf]

1) The province-level and monthly socioeconomic data of China, which is public (open access) and can be downloaded from the link http://www.stats.gov.cn/. The data includes socioeconomic information such as seasonal total GDP, electric power consumption, and the profit from express and logistics.

2) The province-level COVID-19 data of China, which is public (open access) and can be downloaded from the link https://en.wikipedia.org/wiki/Statistics_of_the_COVID-19_pandemic_in_mainland_China. The data includes the number of COVID-19 cases, recoveries, and deaths for each month and province.

3) The monthly and province-level China e-bike safety data is not public. The dataset is owned by the Research Institute for Road Safety (RIRS) of the China Ministry of Public Security (MPS). The data includes e-bike safety information, such as the number of fatalities, the number of injuries, property damage, the distribution of accident causes. The authors of this paper do not have special privileges in accessing the data. For other researchers interested in this dataset, please contact zhuxinyu@122.cn (Mr. Xinyu Zhu, assistant researcher at RIRS, “122.cn” is the official email domain for the China government) for permission/availability of the dataset; any academic use of the dataset will be welcomed, but it could take weeks for processing/delivery of the data due to authority processes from China MPS.

Since data 3) is owned by the RIRS, we cannot release the original raw data. However, we have contacted the data owner who allowed us to publicize the data that is contained in this supporting package.

Table S1. Variables and Meanings of the Dataset.

Variable	Year	Month	Pro	Num_of_Fatalities
Meaning	The year	The month	Province ID	Number of fatalities
Variable	Num_of_Injuries	Num_of_COVID_log	Num_of_Fatalities_lag12	Num_of_Injuries_lag12
Meaning	Number of injuries	Log-transformed COVID-19 cases	Number of fatalities for the last year	Number of injuries for the last year
Variable	GDP (trillion)	Express (billion)	Power (billion KWH)	Pop (M)
Meaning	GDP	Profit from express/logistics	Power consumption	Population
Variable	Income (K)	Urbanization_Rate	Age1	Age2
Meaning	Average annual income	Urbanization rate	Percentage of age group 1 (15 to 64)	Percentage of age group 2 (over 64)
