# Supplementary material for: Quantifying the impact of COVID-19 on e-bike safety in China via multi-output and clustering-based regression models
Source: PLoS One. 2021 Aug 20;16(8):e0256610. doi: 10.1371/journal.pone.0256610 (PMC8378728; doi:10.1371/journal.pone.0256610)
Supplement: Attachment — Submitted filename: renamed_7e85d.pdf [file pone.0256610.s002.pdf]

Responses to Reviewers for the Revised Manuscript PONE-D-21-13700

Dear Editors:

Please find the enclosed revision of "**Quantifying the impact of COVID-19 on e-bike safety in China via multi-output and clustering-based regression models**" co-authored with Mr. Xingpei Yan and Prof. Zheng Zhu. We would like to thank the two anonymous reviewers for their highly constructive comments and suggestions, which have helped us much improve the manuscript currently under review. In particular, we have significantly improved our paper with the following highlights:

- The data issue is addressed by preparing Supporting Information that contains minimal required sample data for this study.
- The figure copyright issue is addressed by replacing the previous map with the map under USGS.
- We have rerun the regression models by including five province-level/country-level variables: population, average annual income, urbanization rate, percentage of age group 1 (15 to 64 years old), and percentage of age group 2 (over 64 years old). Both the country-level multi-output regression results and the province-level clustering-based regression results are updated.
- We have added a literature review in Section 2 of the revised manuscript, which includes the overall impact of COVID-19 on travel demand, traffic safety, and logistics, as well as key socioeconomic factors and their impact on travel volume and safety
- We have rewritten the findings of the models and removed some unsupportive statements.

We provide our response to each reviewer and each comment below, followed by an appropriate revision of the manuscript. **All the detailed revisions are marked with yellow highlights in the uploaded PDF manuscript.**

Response to Journal Requirements

1. Please ensure that your manuscript meets PLOS ONE's style requirements, including those for file naming.

Response:

We have followed the links you provided to adjust the manuscript as PLOS ONE's style. We also move necessary footnotes into the main body.

2. Please address the data availability issue.

Response:

Three data sources are used in this paper:

1) The province-level and monthly socioeconomic data of China, which is public (open access) and can be downloaded from the link http://www.stats.gov.cn/. The data includes socioeconomic information such as seasonal total GDP, electric power consumption, and the profit from express and logistics.

2) The province-level COVID-19 data of China, which is public (open access) and can be downloaded from the link https://en.wikipedia.org/wiki/Statistics_of_the_COVID-19_pandemic_in_mainland_China. The data includes the number of COVID-19 cases, recoveries, and deaths for each month and province.

3) The monthly and province-level China e-bike safety data is not public. The dataset is owned by the Research Institute for Road Safety (RIRS) of the China Ministry of Public Security (MPS). The data includes e-bike safety information, such as the number of fatalities, the number of injuries, property damage, the distribution of accident causes. The authors of this paper do not have special privileges in accessing the data. For other researchers interested in this dataset, please contact zhuxinyu@122.cn (Mr. Xinyu Zhu, assistant researcher at RIRS, "122.cn" is the official email domain for the China government) for permission/availability of the dataset; any academic use of the dataset will be welcomed, but it could take weeks for processing/delivery of the data due to authority processes from China MPS.

Since data 3) is owned by the RIRS, we cannot release the original raw data. However, we have contacted the data owner who allowed us to publicize some processed data used in this paper. In this manner, we share the data from 2019 to 2020 as a minimal anonymized data set. Please find the data and introduction in the Supporting Information

3. Please address the copyright issue of Figure 1 in the previous manuscript.

Response:

We have removed the original **Fig 1** and built a new figure (**Fig 2** in the revision) based on screenshots from the USGS National Map Viewer, which is shown as follows:

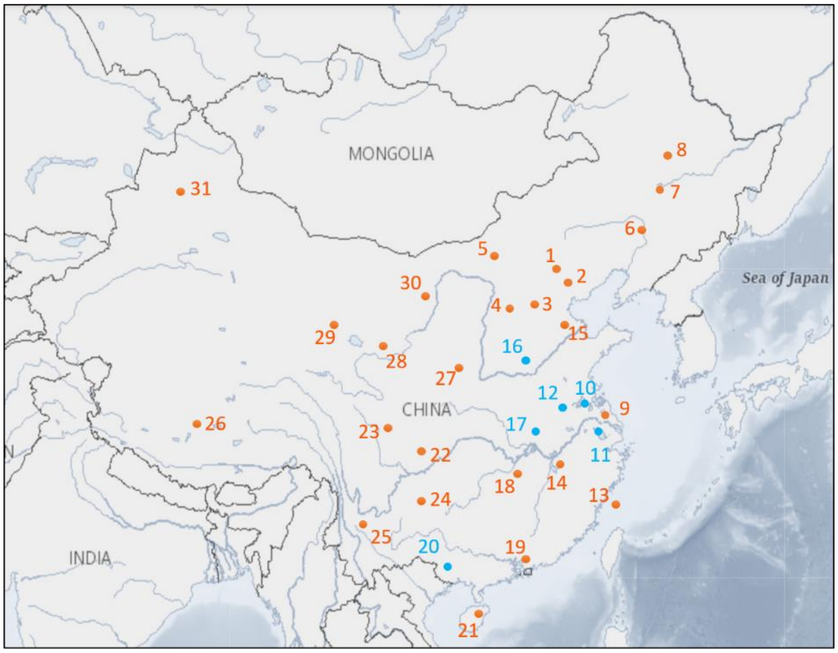

Fig 2. Map of Provinces and Municipalities in China.

Response to Reviewer 1

Reviewer #1: The authors provide an interesting study on analyzing the impacts of COVID-19 on e-bike safety in China. By adding/removing COVID-19 covariates, they show that e-bike safety is sensitive to COVID-19. Two clusters of provinces with different sensitivities to COVID-19 are found based on the clustering-based regression method. The topic is timely and the method is sound. Please address the following comments.

Response:

Thank you for your overall positive comments. We have carefully addressed your comments for major and minor improvements, as shown below.

1) In Table 1, it seems the max monthly number of fatalities/injuries is only 62/520 for a province. The number seems small since the population and area of a China province are huge. I am not judging the data, but please provide a sufficient explanation on why the numbers are so small? Are they representative?

Response:

Thank you for this comment. The data is obtained from the Research Institute for Road Safety (RIRS) of the China Ministry of Public Security (MPS). We have added the following statements about the total number of fatalities in **Section 3.1** to make it more reasonable:

"The total numbers of e-bike fatalities and injuries during the four years in China are 11,137 and 88,331, respectively. Generally, injuries are recorded by the police upon incident calls, but provinces may have different standards for counting injuries."

2) The results need more explanation. In Table 4, the coefficients of "seasonal GDP" and "logit profit" for cluster 2 are negative. The authors claim that "the main reason could be due to the difference in economic structure and people's commute behavior of the two clusters". Please provide evidence on this. Since readers are not familiar with many provinces in China, more background information in terms of e-bike production/sale/use, economic, population, and geographical is necessary.

Response:

Thank you for this constructive comment. First, we have deleted the statement "the main reason could be due to the difference in economic structure and people's commute behavior of the two clusters". Second, we have rerun the regression models by including five province-level/country-level variables: population, average annual income, urbanization rate, percentage of age group 1 (15 to 64 years old), and percentage of age group 2 (over 64 years old). Both the country-level multi-output regression results and the province-level clustering-based regression results are updated. Please refer to the highlighted paragraphs and **Table 1**, **Table 2**, **Table 3**, and **Table 4** in **Section 5** for details. Moreover, we also include some descriptions on e-bike production/sale in **Section 5.2**:

"Furthermore, the usage of e-bikes in provinces in cluster 2 is generally high. For instance, people in

Guangxi province have a large demand for e-bike travel because e-bikes are more economical than over modes concerning the relatively low economic level; Jiangsu province is the largest producer and solder for e-bikes in China. Such information can be obtained by googling the keywords.”

3) Following the previous comment, the policies on e-bike usage and COVID-19 control shall also be different across provinces. Will this make an impact on the results of this study? The authors need to discuss this point and obtain more insights. The author also needs to provide some discussions about how the research findings can help improve traffic safety and epidemic control.

Response:

Thank you for this helpful comment. We have reviewed e-bike policies in different provinces but there is no obvious evidence on how they affect our findings. To address your concerns, we add a paragraph by the end of **Section 5** about the meaning of our research findings:

“To this end, we have found significant negative impacts of COVID-19 on the numbers of e-bike fatalities and injuries at both the country-level and the clustering-based province-level in China. The fact could be caused by the decline of travel demand due to lock-down policies and the people’s panic about the pandemic [19-24]. With the progress of vaccination campaigns, people’s travel demand could recover together with e-bike safety issues in the post-pandemic world. Concerning existing and upcoming safety issues, some provinces, such as Guangxi and Jiangsu, have already implemented e-bike “safe-riding policies” (e.g., wearing a helmet is mandatory) in middle and late 2020. Our findings suggest that encouraging the express/logistics industry can be a promising way to control e-bike safety accidents. Furthermore, e-bike safety problems could be relieved when people get more income.”

4) The authors mention that "30% of road traffic fatalities/injures were caused by e-bike accidents". Is it possible to put a pie chart about the percentage of different traffic accidents in China, this could be interesting and highlight the authors' statement?

Response:

Thank you for this comment. In the revision, we have added a new **Fig 1** that shows the distribution of traffic fatalities and injures in 36 major cities in China during 2019:

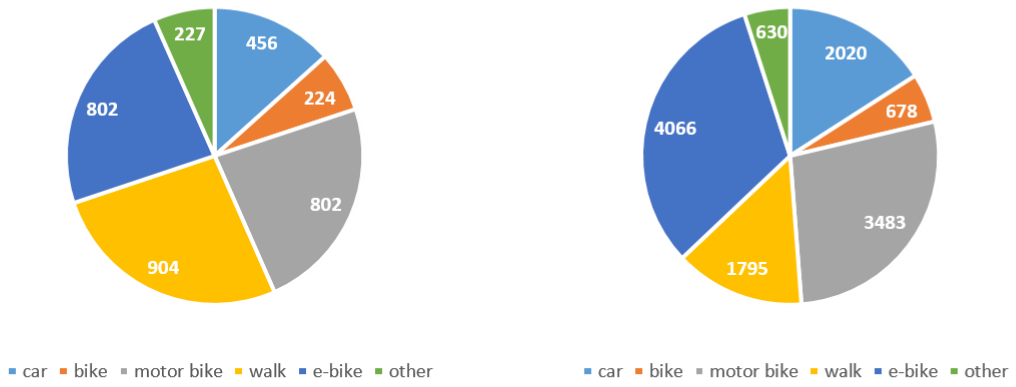

(a) the number of fatalities *(b) the number of injuries*

Fig 1. The Statistics of Traffic Accidents in 36 Major China Cities in 2019.

5) Please improve the language, now there are some typos and grammar errors.

Response:

Thank you for raising this important issue. We have proofread the paper carefully with the help of native speakers. Now the writing quality of the revised paper has been improved. Thank you again for helping us improve this paper.

Response to Reviewer 2

Reviewer #2: The paper examines the relationship between the number of COVID-19 cases and e-bike safety metrics such as number of fatalities and injuries. This is achieved through fusing multiple data sources and adopting a multi-output clustering-based regression analysis at the national and provincial levels. By interpreting the results of the regressions, the authors conclude that a higher number of covid-19 cases has a negative effect on the number of e-bike fatalities and injuries, and that this effect is more pronounced for the cluster of provinces with more e-bike fatalities and injuries. While the specific subject matter (COVID-19's impact on e-bike safety) is novel, the paper can be improved by addressing the following issues:

Response:

Thank you for your overall positive comments. We have carefully addressed your comments for major and minor improvements, as shown below.

(1) The paper would benefit greatly from a thorough literature review of how COVID-19 affects travel behavior and specifically traffic safety. This will help authors to compare their findings on e-bikes to other modes of transportation like bicycles or motor vehicles. Also, the impact of COVID-19 on logistics and delivery businesses can be examined as well.

Response:

Thank you for this constructive comment. We have added a literature review in **Section 2** of the revised manuscript, which includes the overall impact of COVID-19 on travel demand, traffic safety, and logistics:

"Before discussing the data, methodology, and analysis results, we present a review of the impact of COVID-19 on travel demand and traffic safety, as well as the key features/covariates used for traffic safety regression studies. The review identifies the contributions of this paper as a supplement to the existing literature on COVID-19 and traffic safety-related studies.

Since the breakout of COVID-19 in Hubei, China in late 2019, there have been three world-wide infection waves of the epidemic: 1) the first wave peaked between early spring and summer in 2020 when countries started to implement lock-down strategies; 2) the second wave witnessed the rebounding of infection during October to the end of 2020 as some countries relaxed social distance policies; 3) the third wave began in early 2021 along with the start of vaccination campaigns since some countries loosed lock-downs and travel restrictions [15]. The threat from the virus and lock-down policies significantly impact people's travel demand and travel patterns. The most heavily damaged transportation sector is air transport, which suffered from a decline of 50% seats (around 2.9 billion passengers and 390 billion USD) [16]. Similarly, rail transport, another essential long-distance travel mode, has experienced 20% to 30% annually passenger loss in different regions such as Europe, the US, and Asia [17-18]. Apart from long-distance mobility, it was worth noting that both land-borne and maritime freight transport have met certain degrees of financial crisis in the phase of pandemic, while e-commerce companies, which focus on business-to-consumer sections, such as UPS, FedEx, and Amazon became the winner [15]. The findings

indicate a notable decline in people's travel/social/shopping/ activities.

We examine how the pandemic has been changing people's daily travel patterns in terms of the overall trip demand and the usage of different ground transport modes. Based on long-term mobile device location data in the US, the University of Maryland research team showed that the pandemic itself has dramatically reduced people's number of trips and trip mileage, especially in states in a serious infection situation; while the pure impact of lock-down policies signed by governments is much smaller [19-21]. Using the same dataset, Xiao et al. found that people's trip duration has notably decreased after the national call [22]. Similar evidence on the decline of travel frequency and durations are found in other regions, such as Asia and Europe [23-24]. Besides changes in travel frequency and durations, it was observed that people tend to choose private cars or bikes rather than subway, buses, or taxis (ride-hailing services) to stay away from the gathering and a high infection chance in many places, such as Germany, Canada, Scotland, and China [25-28].

In addition to reshaping people's travel demand and habits, the pandemic and lock-down policies also affect traffic safety. It was found that as the number of trips dropped greatly and the number of roadway accidents decreased significantly in the US [29-31]; however, the fatality rate during traffic accidents increased by around 14% in early 2020 compared with 2019 [32]. Using traffic flow and incident data in Greece, Katrakazas et al. found that the lock-down policy caused more vehicle accidents since people drove faster with fewer vehicles on roadways [33]; over-speed behaviors during the epidemic were also found in the UK and France [34]. The ETSC reported a notable decrease in the numbers of incidents and fatalities during the pandemic in some European countries, such as Spain, Italy, Finland, and Germany, which was largely due to the reduction of trips [33-34]."

(2) Another aspect of a thorough literature review should focus on how socioeconomic factors affect travel volume and safety. This would help justify the choice of predictor variables in the paper, and hopefully support statements like in page 4 line 20-22: "the unbalance in socioeconomic levels brings heterogeneity in the number of COVID-19 cases and the e-bike safety". Currently, the variable choices are not well justified. Some theoretical foundation is needed here. Other census statistics are also worth trying (even though they are mostly static): population, age, income level, urbanization rate, motorization rate, etc.

Response:

Thank you for this helpful comment. First, to continue with the previous response, we have added a paragraph in the literature review section about key socioeconomic factors and their impact on travel volume and safety:

"According to the aforementioned review, there are still insufficient studies about the impact of COVID-19 on roadway safety; and most safety analyses were conducted based on car-related crashes, while nonmotorized travel modes (e.g., walk, bike, and e-bike) have drawn little attention. It was estimated that cyclists and pedestrians would enjoy a much safer trip since the pandemic has led to certain reductions in vehicle volume on roadways [35]. With e-bike accident data before and after the pandemic, we try to

fill a research gap by analyzing how COVID-19 impacts e-bike safety in China. To conduct a solid empirical study, we fuse the accident dataset with public socioeconomic features that were highly correlated with traffic/bike/e-bike safety in the literature. For example, population and population density generally indicate the overall travel demand that can be positively related to the number of accidents [36-37]; GDP and economic-related features reflect people's activity and travel frequency [36-37]; income tends to have positive effects on car travel demand [38]; age group and gender is associated with travel mode and driving/riding behavior [39-40]; urbanization level partially shows travel density in the study area [37, 41]."

Second, we have deleted the statement "the unbalance in socioeconomic levels brings heterogeneity in the number of COVID-19 cases and the e-bike safety". We added a new sentence to describe the e-bike safety data in **Section 3**:

"The total numbers of e-bike fatalities and injuries during the four years in China are 11,137 and 88,331, respectively. Generally, injuries are recorded by the police upon incident calls, but provinces may have different standards for counting injuries."

Third, we have rerun the regression models by including five province-level/country-level variables: population, average annual income, urbanization rate, percentage of age group 1 (15 to 64 years old), and percentage of age group 2 (over 64 years old). Both the country-level multi-output regression results and the province-level clustering-based regression results are updated. Please refer to the highlighted paragraphs and **Table 1**, **Table 2**, **Table 3**, and **Table 4** in **Section 5** for details.

Last, we also include some descriptions on e-bike production/sale in **Section 5.2**:

"Furthermore, the usage of e-bikes in provinces in cluster 2 is generally high. For instance, people in Guangxi province have a large demand for e-bike travel because e-bikes are more economical than over modes concerning the relatively low economic level; Jiangsu province is the largest producer and solder for e-bikes in China. Such information can be obtained by googling the keywords."

(3) K-means typically requires the data (x and y) to be normalized. It is not clear whether this is done. Based on Table 3, it seems the data is not properly normalized. In addition, the K-means is applied to 31 provinces (not 1000+ records), implying that the panel data is somehow aggregated at the provincial level. This needs more explanation.

Response:

Thank you for this comment. First, we have already normalized the data before clustering. To explain it clearly, we have revised the description of the clustering-based regression:

"The clustering task is conducted via the K-means algorithm [36]. Before clustering, one needs to normalize $\boldsymbol{x}_t^j$ *and* $\boldsymbol{y}_t^j$ *based on the general 0-1 approach, i.e.,* $\tilde{x}_{t,m}^j = \frac{x_{t,m}^j - \min_{j',t'}\left(x_{t',m}^{j'}\right)}{\max_{j',t'}\left(x_{t',m}^{j'}\right) - \min_{j',t'}\left(x_{t',m}^{j'}\right)}$. *Let* $\tilde{\boldsymbol{x}}_t^j$, $\tilde{\boldsymbol{y}}_t^j$, *and* $\widetilde{\boldsymbol{W}}^j$ *denote the normalized variables and datasets, and let* $(\boldsymbol{p}_k, \boldsymbol{q}_k)$ *denote the centroid of these normalized data records, where* $\boldsymbol{p}_k$ *and* $\boldsymbol{q}_k$ *represent centroids of predictive variables and target variables, respectively. By randomly taking* K *centroids* $\{(\boldsymbol{p}_1, \boldsymbol{q}_1), (\boldsymbol{p}_2, \boldsymbol{q}_2), \dots, (\boldsymbol{p}_K, \boldsymbol{q}_K)\}$ *for initialization, the K-means algorithm iteratively proceeds the following two steps and will terminate once the centroids converge.*

- ***Assignment Step****. Traverse the subpopulations, i.e.,* $j \in \{1,2, \dots, J\}$*; for* $\widetilde{\boldsymbol{W}}^j$*, assign all the samples to class* k *if the sum of the squared Euclidean distances to centroid* $(\boldsymbol{p}_k, \boldsymbol{q}_k)$ *is the minimum for* $k \in \{1,2, \dots, K\}$*:*

$$L(j,k) = \sum_{t=1}^{T} \left\| \left(\tilde{\boldsymbol{x}}_t^j, \tilde{\boldsymbol{y}}_t^j\right) - (\boldsymbol{p}_k, \boldsymbol{q}_k) \right\|^2 \quad (3)$$

$$\boldsymbol{C}_k = \left\{ \left(\tilde{\boldsymbol{x}}_t^j, \tilde{\boldsymbol{y}}_t^j\right) \in \widetilde{\boldsymbol{W}}^j : L(j,k) \le L(j,k'), k' \in \{1,2, \dots, K\}, j \in \{1,2, \dots, J\} \right\} \quad (4)$$

where $L(j,k)$ *denotes the sum of squared Euclidean distances from each sample in subpopulation* j *to the centroid of cluster* k*.*

- ***Updating Step.*** *Traverse the clusters, i.e.,* $k \in \{1,2, \dots, K\}$*, compute the new centroids based on the current clustering results:*

$$\boldsymbol{p}_k = \frac{\sum_{\widetilde{\boldsymbol{W}}^j \in \boldsymbol{C}_k} \sum_t^T \tilde{\boldsymbol{x}}_t^j}{\sum_{\widetilde{\boldsymbol{W}}^j \in \boldsymbol{C}_k} N^j}, \boldsymbol{q}_k = \frac{\sum_{\widetilde{\boldsymbol{W}}^j \in \boldsymbol{C}_k} \sum_t^T \tilde{\boldsymbol{y}}_t^j}{\sum_{\widetilde{\boldsymbol{W}}^j \in \boldsymbol{C}_k} N^j} \quad (5)$$

After clustering, the original dataset is divided into K disjointed datasets and data records in the same cluster share a more similar pattern to each other than to those in other clusters. Multi-output regression models are fitted for each specific dataset, which has the following model formulation:

$$\boldsymbol{y}_t^j = \boldsymbol{x}_t^j \sum_{k=1}^{K} I_{j,k} \boldsymbol{w}_k + \boldsymbol{e}_t \quad (6)$$

where $I_{j,k}$ *denotes the indicator whether subpopulation* j *belongs to cluster* k *(i.e., if* $\boldsymbol{D}^j \subseteq \boldsymbol{C}_k$, $I_{j,k} = 1$*; else,* $I_{j,k} = 0$*),* $\boldsymbol{w}_k$ *denotes the coefficient matrix for cluster* k*. For each cluster, we estimate* $\boldsymbol{w}_k$ *by minimizing its regression loss (i.e., Eq. (2))."*

The K-means is applied to the monthly panel data rather than the aggregate data. That is, we use 31*48 data records in the K-means algorithm; for the 48 records belonging to a specific province, their cluster label shall be the same. We call this a subpopulation-based clustering such that data records from a specific province belong to the same subpopulation; we group different subpopulations into clusters to capture

homogenous regression attributes within clusters (groups of provinces) and heterogeneous regression attributes across clusters. We have clarified this in Section 4.2:

"For the province-level analysis, we continue with the multi-variate and multi-output settings. The time-series dataset $\boldsymbol{W}$ *is collected from* J *disjoint subpopulations (i.e., provinces and municipalities in this paper) and there are* T *samples (i.e., time periods) for each subpopulation, i.e.,* $\boldsymbol{W} = \cup_{j=1}^{J} \boldsymbol{W}^j$ *and* $\boldsymbol{W}^j = \{(\boldsymbol{x}_1^j, \boldsymbol{y}_1^j), (\boldsymbol{x}_2^j, \boldsymbol{y}_2^j), \dots, (\boldsymbol{x}_T^j, \boldsymbol{y}_T^j)\}$ *for* $j \in \{1,2, \dots, J\}$*. We group subpopulations into* K $(K \leq J)$ *clusters such that all the samples from one subpopulation shall belong to the same cluster. Let* $\boldsymbol{C}_k$, $k \in \{1,2, \dots, K\}$, *denote the subset of data records that belong to cluster* k*. Any two clusters* k *and* k' *are disjoint, i.e.,* $\boldsymbol{C}_k \cap \boldsymbol{C}_{k'} = \emptyset$*, for* $k \neq k'$ *and* $k, k' \in \{1,2, \dots, K\}$*; the union of all clusters is the full dataset, such that* $\cup_{k=1}^{K} \boldsymbol{C}_k = \boldsymbol{W}$*. In this manner, we utilize the province-level panel data for clustering based on their similarity in e-bike safety and socioeconomic metrics and the cluster labels of data records from the same province are identical."*

(4) Some of the "conclusions" in Section 4.1 (page 9 line 13-20) and Section 4.2 (page 10 line 21-32) are mostly speculative and not well supported by model results. The core of the issue lies in how to disentangle the effects of change in overall travel demand vs change in "unsafe riding habits". Related to (2), a literature review and some theoretical foundation can be helpful here. If this cannot be addressed, then the authors should be very careful about their conclusions.

Response:

Thank you for raising this critical issue. We agree with you that "we cannot disentangle the effects of change in overall travel demand vs change in unsafe riding habits". We have removed some of the previous statements.

The rewritten statements of **Section 5.1** is:

"Concerning both goodness-of-fit and simplicity (i.e., less collinearity among predictive variables), we select the COVID-19 model with $\lambda_1 = 1.0$ *for quantitative analysis. The lagged number of fatalities and lagged number of injuries are positively related to the outputs, which is consistent with the profile plot of Fig 3. The log-transformed number of COVID-19 cases has a strong negative impact on the numbers of fatalities (a coefficient of -11.77) and injuries (a coefficient of -50.85). An intuitive explanation could be that people tend to make much fewer trips during the pandemic. Although there are no direct observations on e-bike travel demand, the results are consistent with findings in other modes [29, 33, 35]. Power consumption and seasonal GDP, which reflect living, social, and productivity levels, have positive influences on the numbers of fatalities and injuries [36]. Due to collinearity between socioeconomic features, we find the coefficients of express/logistics profit and percentage of age group 2 are zero (i.e., the later variable is negatively related to GDP because a higher percentage of aged people means a lower labor level). Average income has small negative coefficients, which could result from the negative relationship between income and e-bike usage (or positive relationship between income and driving/taxi) [47]. Given the linear relationship between socioeconomic and e-bike safety metrics, we are still unclear whether unsafe riding habits are under control or not during these years [48], and more variables are*

needed to conduct further analyses. In summary, COVID-19 relieves e-bike safety issues in China, possibly due to the decline of overall living and social activities and e-bike travel demand."

The rewritten statements of **Section 5.2** is:

*"Following the clustering-based multi-output regression approach introduced in **Section 4**, we try different combinations of λ_1 and λ_2 for province-level analyses for the identified two clusters. After balancing goodness-of-fit and model simplicity, we select $\lambda_1 = 0.1$ and $\lambda_2 = 0$. The regression results are shown in **Table 4**. First, the impact of (the log-transformed) number of COVID-19 cases on cluster 2 (i.e., -1.660/-21.67 for fatality/injury) is notably higher than cluster 1 (i.e., -0.012/-0.325 for fatalities/injuries). Second, we note the coefficients of population are negative for cluster 2, which is somehow different from previous traffic safety analyses based on small scale (i.e., block-based or zip code-based) populations [37]. This could be caused by the collinearity between population and urbanization rate, which also has negative coefficients for cluster 2. Meanwhile, the two variables have zero coefficients for cluster 1, indicating that e-bike safety is insensitive to rural and urban populations in provinces with a minor safety issue. Third, e-bike safety is found insensitive to the percentage of age group 2 in both clusters. Based on the results in **Table 2**, we know the rate of the aged population is negatively related to e-bike incidents, but the coefficients are dragged to zero with the Lasso regularization. Last, profit from express/logistics has negative coefficients for cluster 2, this could be caused by a huge amount of online shopping activities in these provinces (Zhejiang and Jiangsu are two giant provinces in China for online shopping) that would reduce the demand for travel as well as for e-bike."*

Moreover, we add a paragraph in by the end of **Section 5** about the meaning of our research findings:

"To this end, we have found significant negative impacts of COVID-19 on the numbers of e-bike fatalities and injuries at both the country-level and the clustering-based province-level in China. The fact could be caused by the decline of travel demand due to lock-down policies and the people's panic about the pandemic [19-24]. With the progress of vaccination campaigns, people's travel demand could recover together with e-bike safety issues in the post-pandemic world. Concerning existing and upcoming safety issues, some provinces, such as Guangxi and Jiangsu, have already implemented e-bike "safe-riding policies" (e.g., wearing a helmet is mandatory) in middle and late 2020. Our findings suggest that encouraging the express/logistics industry can be a promising way to control e-bike safety accidents. Furthermore, e-bike safety problems could be relieved when people get more income."

(5) The writing of the paper needs some refinement. Take Section 2 for example:
- Page 4, line 15-16. The following sentence does not seem to make much sense: "For all these variables, the average values are greater than the medium values, indicating that there are 'metropolitan' provinces/municipalities in China."
- Page 4, line 28: "transferred" -> "log-transformed"? Also, Figure 2 needs a legend.
- Page 5, line 3: "increase" -> "increases"

There are other examples like this. A thorough proofread would be very helpful!

Response:

Thank you for raising this important issue. We have proofread the paper carefully with the help of native speakers. Now the writing quality of the revised paper has been improved. We also add a legend to **Fig 3**:

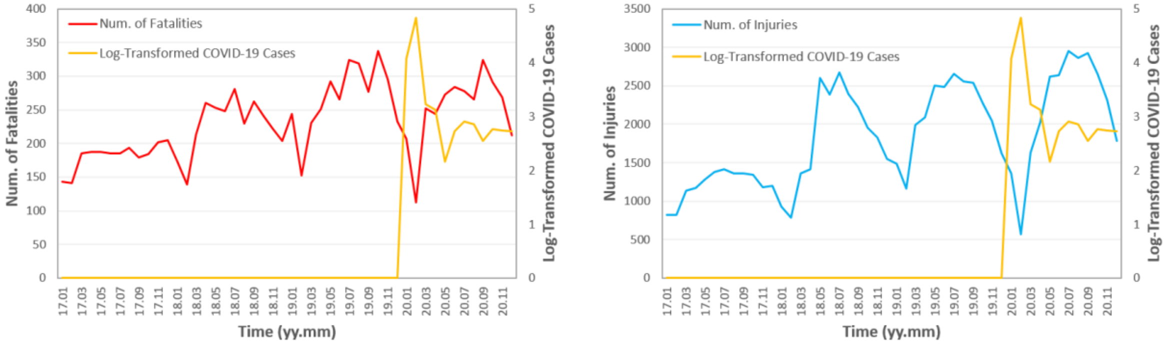

(a) the number of fatalities *(b) the number of injuries*

Fig 3. The Trend of E-Bike Safety in China.”

Thank you again for helping us improve this paper.
