# Supplementary material for: Quantifying the impact of COVID-19 on e-bike safety in China via multi-output and clustering-based regression models
Source: PLoS One. 2021 Aug 20;16(8):e0256610. doi: 10.1371/journal.pone.0256610 (PMC8378728; doi:10.1371/journal.pone.0256610)
Supplement: Attachment — Submitted filename: renamed_61fe4.pdf [file pone.0256610.s003.pdf]

Responses to Reviewers for the Revised Manuscript PONE-D-21-13700

Dear Editors:

Please find the enclosed revision of "**Quantifying the impact of COVID-19 on e-bike safety in China via multi-output and clustering-based regression models**" co-authored with Mr. Xingpei Yan and Prof. Zheng Zhu. We would like to thank the two anonymous reviewers for their highly constructive comments and suggestions, which have helped us much improve the manuscript currently under review. In particular, we have significantly improved our paper with the following highlights:

- We have added boundaries of provinces in Fig 2.
- We proofread the manuscript carefully and the writing has been notably improved.
- We add discussions on policy implications.

We provide our response to each reviewer and each comment below, followed by an appropriate revision of the manuscript. **All the detailed revisions are marked with yellow highlights in the uploaded PDF manuscript.**

Response to Journal Requirements

Please review your reference list to ensure that it is complete and correct. If you have cited papers that have been retracted, please include the rationale for doing so in the manuscript text, or remove these references and replace them with relevant current references. Any changes to the reference list should be mentioned in the rebuttal letter that accompanies your revised manuscript. If you need to cite a retracted article, indicate the article's retracted status in the References list and also include a citation and full reference for the retraction notice.

Response:

We have checked and ensured that the reference list is correct. There is no paper that has been retracted.

Response to Reviewer 1

Reviewer #1: The authors have addressed all my comments. This quality has been improved substantially. I recommend this manuscript for publication.

Response:

Thank you again for helping us improve our paper.

Response to Reviewer 2

Reviewer #2: Overall, the authors did a good job responding to the prior comments. There are only a couple of minor comments:

Response:

Thank you for your overall positive comments. We have carefully addressed your comments for improvements, as shown below.

(1) One of the only remaining issues is the writing. Grammatical errors are still not uncommon. For example, in Literature Review:

- Page 4, Line 22-23, "It was found that as the number of trips dropped greatly and the number of roadway accidents decreased significantly in the US [29-31]"

- Page 5, Line 1-2, "age group and gender is associated with travel mode and driving/riding behavior [39-40]"

- Some wording issues: “lock-down" -> "lockdown"; "over-speed" -> "speeding"

I think the writing can be further improved by another round of proofreading.

Response:

Thank you for this critical comment. We have carefully proofread the manuscript, and the writing issue has been notably improved. Please see the highlighted changes in the revised manuscript.

(2) It is recommended to replace Fig. 2 with a map showing the provincial boundaries.

Response:

Thank you for this comment. We have added provincial boundaries, and the new Fig. 2 is given by

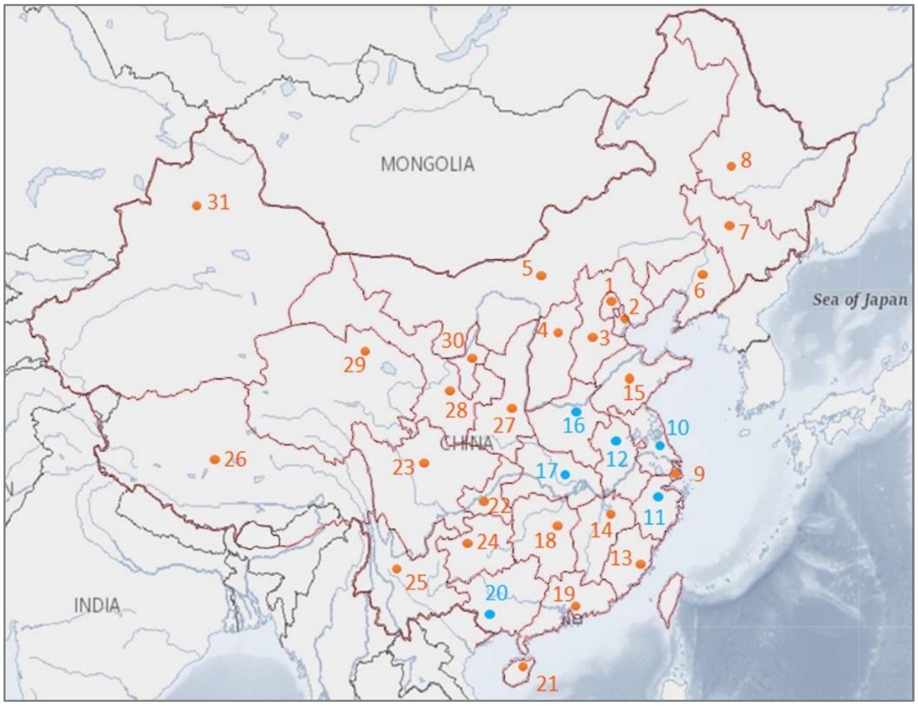

Fig 2. Map of Provinces and Municipalities in Mainland China.

(3) In the conclusion section, the authors may consider to add more discussion on policy implications. Currently, it is not clear how we can use the results of this paper in practices.

Response:

Thank you for this constructive comment. We have added some discussions on policy implications in both **Section 5** and **Section 6**.

In **Section 5**:

"Furthermore, the results in this paper could be helpful in policy implications. Our findings suggest that encouraging the express/logistics industry can be a promising way to control e-bike safety accidents. E-bike safety problems could be relieved when people get more income; alternatively, the promotion of other economical travel modes, such as car sharing, could be an effective way for reducing e-bike demand. In addition, for provinces in cluster 2, transportation agencies need to enhance safety enforcement in rural areas."

In **Section 6**:

"The regression results can be helpful in policy implications that further reduce e-bike safety issues; for instance, the government could encourage the express/logistics industry to decrease e-bike demand and e-bike accidents."

Thank you again for helping us improve our paper.
